# Unusual coexistence of restrictive heart disease and Kallmann syndrome: a case report

**DOI:** 10.1186/s43044-024-00479-1

**Published:** 2024-04-18

**Authors:** Ghali Bennani, Soukaina Zahri, Mohamed Khaldi, Ghali Benouna, Abdenasser Drighil, Rachida Habbal

**Affiliations:** Department of Cardiology, University Hospital of Ibn Rochd, Lotissement Lina Villa 46 Sidi Maarouf, Casablanca, Morocco

**Keywords:** Hypogonadotropic hypogonadism, Amenorrhea, Anosmia, Restrictive cardiomyopathy, Case report

## Abstract

**Background:**

Kallmann–Morsier syndrome is a rare disease characterized by the association of congenital gonadotropic deficiency and anosmia or hyposmia. The cardiac manifestations associated with this syndrome are little known. Through this case, we will characterize the cardiac involvement of this disease in the light of what is already described in the literature.

**Case presentation:**

We report the case of a young patient who presented with a picture of cardiac decompensation revealing restrictive heart disease. In her exploration, she was found to have primary amenorrhea, leading to the diagnosis of Kallmann syndrome. Medical treatment was optimized for the management of her cardiac decompensation as well as hormonal replacement treatment for her delayed puberty and growth.

**Conclusions:**

Cardiac manifestations in Kallmann–Morsier syndrome are few reported in the literature, and restrictive heart disease is uncommon with no cases report till now. This association suggests a possible common genetic origin that should be explored in the future.

## Background

Kallmann–Morsier syndrome (KS) is a rare (1/10,000) disease of neuronal development defined by the association of congenital gonadotropic deficiency and anosmia or hyposmia, linked to hypoplasia or aplasia of the olfactory bulbs [1, 2]. It is characterized by both genetic and phenotypic heterogeneity, often discovered during the exploration of delayed puberty. The prevalence is underestimated in girls due to a less obvious female diagnosis. Genetically, two forms are described: the familial form and the sporadic form, which remains the most common [3]. Until now, eight genes have been identified; however, no mutation in one of these eight genes is found in approximately 60 to 65% of patients. Therapeutically, hormonal treatment aims to start puberty and maintain secondary sexual characteristics. This clinical case reports our experience in the management of an unknown heart disease discovered in a 19-year-old patient with KS syndrome.

## Case presentation

A 19-year-old female patient, single, with no particular history, admitted for global cardiac decompensation with a productive cough and dyspnea at rest for 10 days. On admission, the patient presented signs of heart failure such as crackles rales at the bases, turgidity of the jugular veins and edema of the lower limbs. There was also a muffled heartbeat. The rest of the examination found a patient with delayed height and weight (body mass index at 16 kg/m2), absence of development of secondary sexual characteristics, as well as a notion of unexplored primary amenorrhea and associated anosmia.

**Fig. 1:**
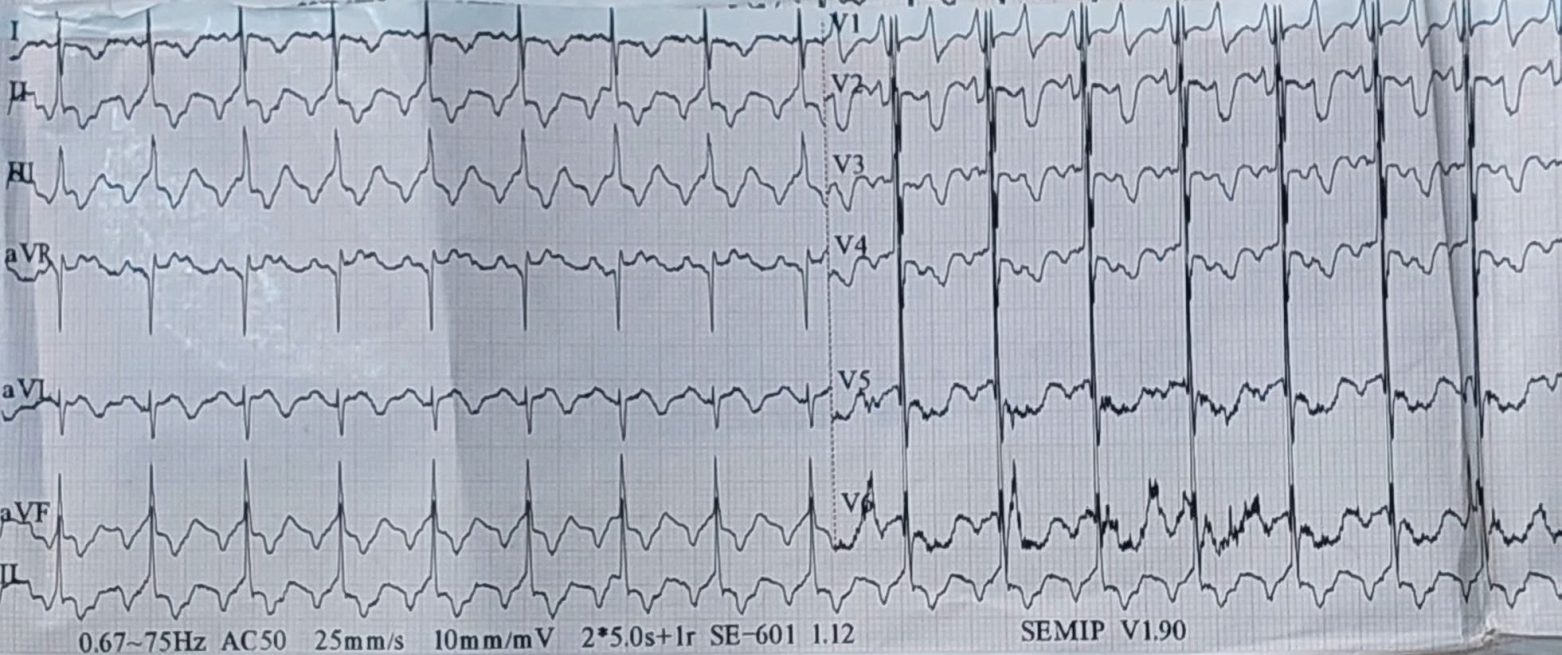
EKG

**Fig. 2:**
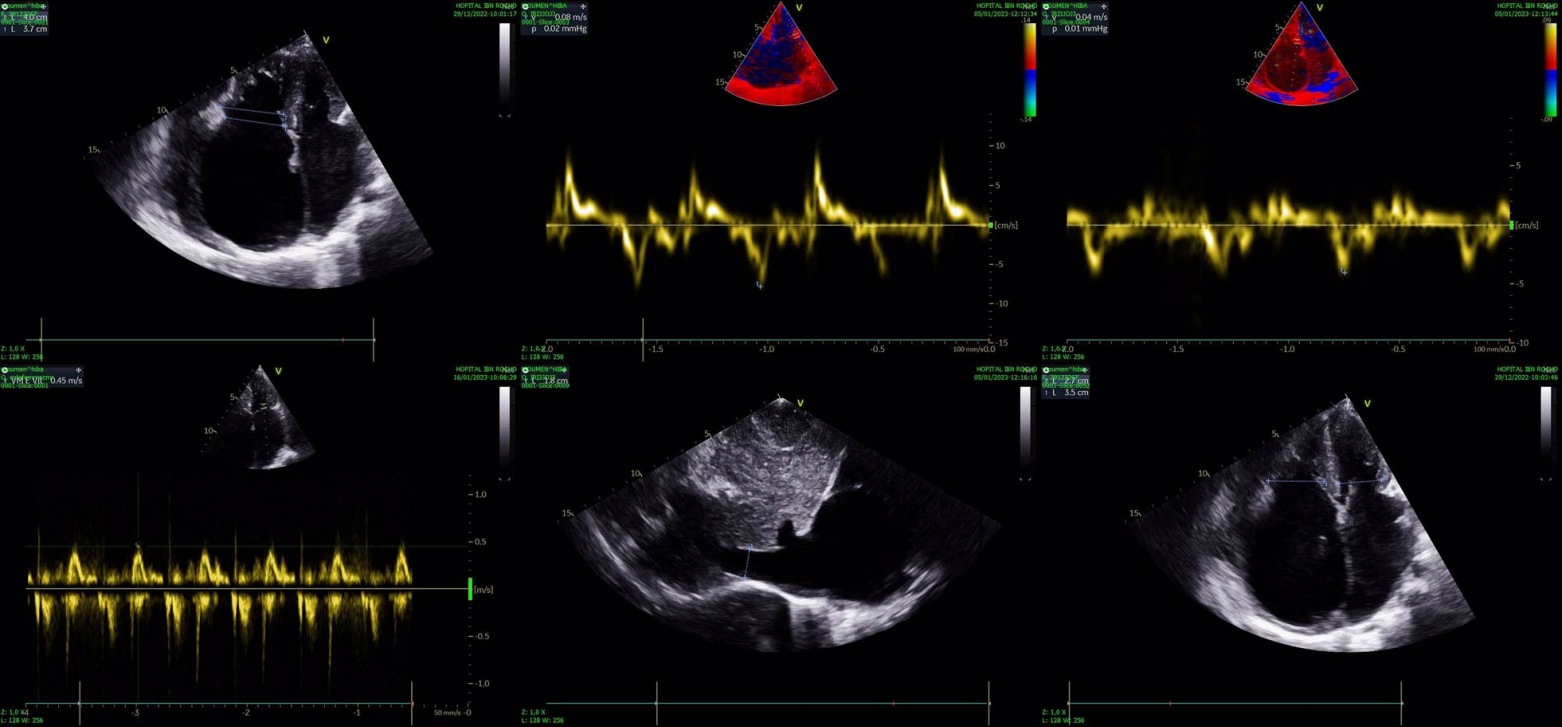
Signs suggestive of restrictive cardiomyopathy

**Fig. 3:**
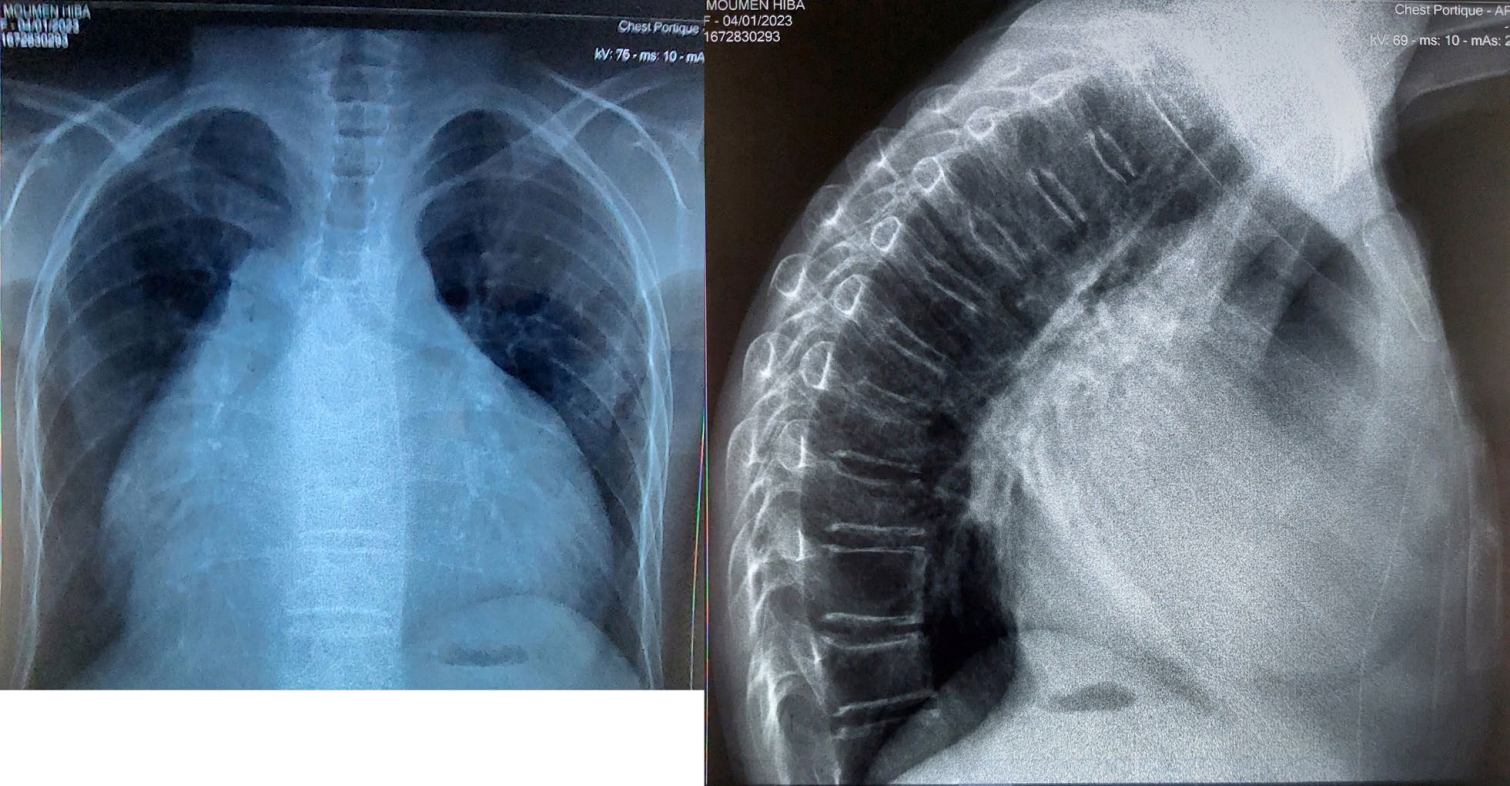
Chest X-ray front and profile

On the electrocardiogram (EKG), a flutter was noted at 112 beats per minute (Fig. 1). On transthoracic echocardiogram (TTE), we had a non-hypertrophied left ventricle with good contractility and strain altered to − 10%, biatrial dilation, small pericardial effusion, no mitroaortic valve disease and no variations in respiratory flow, a mitral profile restrictive, altered E' waves, dilated and altered right ventricle, elevated pulmonary pressures with dilation of the inferior and suprahepatic vena cava (Fig. 2). All these signs suggestive of restrictive cardiomyopathy, chronic constrictive pericarditis being less likely given the absence of thickening of the pericardium. On chest X-ray, we found cardiomegaly without calcifications of the pericardium (Fig. 3).

**Fig. 4:**
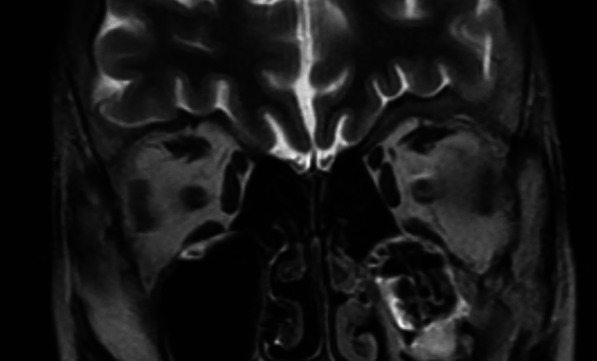
Hypothalamic–pituitary MRI showing hypoplasia of the olfactory bulbs

**Fig. 5:**
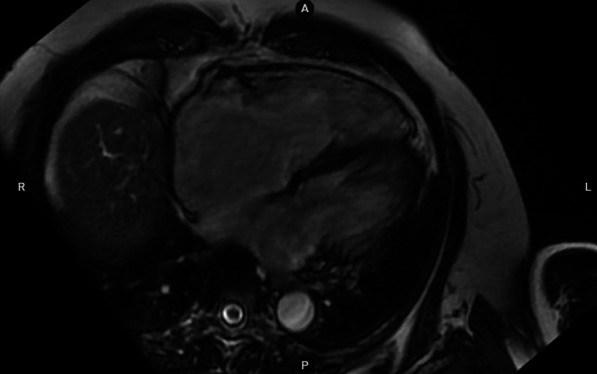
MRI cardiac

On biology, there was hyponatremia at 124 mEq/L, brain natriuretic peptide (BNP) at 737 [< 18.4] pg/ml, good renal function and no hypereosinophilia. Hypogonadotropic hypogonadism was observed: follicle-stimulating hormone FSH = 0.84mIU/ml (2–10), luteinizing hormone LH = 0.13mIU/ml (0.5–5), estradiol < 10 pg/ml. The assessments looking for overload or infiltrative disease (hemochromatosis, Fabry, amyloidosis) came back negative. Hypothalamic–pituitary MRI was performed showing hypoplasia of the olfactory bulbs (Fig. 4). Cardiac catheterization was carried out, finding no dip tray appearance, nor equalization of pressures in cardiac cavities. The cardiac biopsy was not performed, being unavailable in our center. Cardiac MRI showed dilation of the right cavities, without signs of pericardial thickening or infiltration or overload (Fig. 5). In the genetic study, no mutations were found and the karyotype was normal. Exploration of the other hypothalamic–pituitary axes did not reveal any insufficiency and renal ultrasound was normal.

Treatment for heart failure based on diuretic, beta-blocker and ACE inhibitor was given to the patient, in addition to hormone replacement therapy based on Estradiol with a daily oral dosage of 2 mg, with the appearance of regular menstrual cycles and no changes of her heart condtion at follow-up. The patient has good compliance and tolerance of her treatment. No recurrence and no side effects have been reported.

## Discussion

Congenital hypogonadotropic hypogonadism is caused by decreased release of gonadotropins in the hypothalamus and is classified into several phenotypes. KS is an example and is the most common syndrome associated with congenital anosmia [1]. Hypogonadism, which is the mechanism involved, is secondary to abnormalities in neuronal development affecting the prenatal migration of GnRH neurons, whereas anosmia is secondary to atrophy of the olfactory bulbs and/or lobes [2]. Its clinical picture is characterized in most cases by delayed pubertal development and is confirmed by hormonal dosage and hypothalamic–pituitary MRI focused on the olfactory bulbs, as for our patient.

It is also not uncommon to find other clinical manifestations, particularly cardiac in these patients. Cases of hypogonadotropic hypogonadism associated with Wolf–Parkinson–White syndrome have been reported in the literature [3, 4]. Recent studies have also shown that testosterone deficiency can induce metabolic disorders such as hyperglycemia and the progression of atherosclerotic disease, particularly coronary artery disease [5, 6]. Gould and Reddy reported a patient with KS and second-degree heart block and atrioventricular node conduction delay, suggesting that there may be a link between KS and cardiac abnormalities associated with GnRH [7]. Other studies have reported the presence of ebstein disease, right aortic arch and left subclavian stenosis [8, 9].

In our patient, we find the existence of a restrictive cardiomyopathy, which is also a rare pathology with a strong genetic component. Given the rarity of the two pathologies, their association may be a simple coincidence or an attack of several common genes involved in their pathogenesis [10, 12]. Furthermore, studies have reported the presence of GnRH receptors in the human heart, suggesting effects on the cardiovascular system [11, 12].

Genetically, KS is featured in several disorders related to genes, with a high degree of genetic variation, and only approximately 40% of Kallmann syndrome is caused by known genetic mutations: KAL1 and FEZF1 [1, 2]. In our patient, the genetic study did not find anything particular.

The rarity of the combination of these conditions makes it difficult to potentially identify a possible common genetic mutation, but genetic testing/sequencing could produce candidate genes that could indicate possible common genetic links.

## Conclusions

Advances in genetics have allowed us to better understand pathologies such as KS or restrictive cardiomyopathy and may reveal possible genetic pathways that could explain the rare association between these two pathologies observed in the current patient. However, the genetic study is not always conclusive, given the complex nature of the neuroendocrine system and the genes involved. Further research into the molecular basis of the disease and the various signaling pathways involved will help develop early screening and diagnosis to ensure better management.

## Data Availability

Data supporting the study results can be provided followed by request sent to the corresponding author’s e-mail.

## Abbreviations

MRI: Magnetic resonance imaging
GnRH: Gonadotropin-releasing hormone
KS: Kallmann syndrome
EKG: Electrocardiogram
TTE: Transthoracic echocardiogram
BNP: Brain natriuretic peptide
FSH: Follicle-stimulating hormone
LH: Luteinizing hormone
ACE: Angiotensin-converting enzyme
